# Editorial: Stars and rising stars in pediatric endocrinology: 2022

**DOI:** 10.3389/fendo.2024.1384549

**Published:** 2024-03-18

**Authors:** Benjamin Udoka Nwosu

**Affiliations:** Department of Pediatrics, Zucker School of Medicine at Hofstra/Northwell, Chief of Endocrinology, Cohen Children’s Medical Center of New York, Director, Children’s Diabetes Center, Lake Success, NY, United States

**Keywords:** continuous glucose monitor, wolfram (DIDMOAD) syndrome, disorders of sex development (DSD), COVID - 19, hyperlipidemia, type 1 diabetes, honeymoon phase, multiple endocrine neoplasia

Endocrinology is an evolving field, and this Research Topic, *Stars and Rising Stars in Endocrinology*, was offered to attract the works of the best brains in the field. The Rising Stars are mid-level to upper-level clinicians and scientists who are making their mark in Endocrinology. This *Editorial* covers research work from diverse areas of endocrinology that range from the genetics of rare endocrine disorders to novel applications of diabetes technology and a new theory of dyslipidemia in endocrinology. Each of these works is forward-leaning and promises a wider application in endocrinology. From the newly proposed theory of hyperlipidemic memory of type 1 diabetes to the use of whole-exome sequencing to identify a novel mutation causing a disorder of sexual development, and the use of transcriptomic data to diagnose growth hormone (GH) deficiency in children, this Research Topic contains innovative works that will shape the future of endocrinology.

Several articles focused on improving the diagnostic approaches to endocrine disorders. Garner et al. report on innovative techniques to improve the diagnosis of GH deficiency (GHD) in children with short stature. Currently, the diagnosis of GHD in children is imprecise as the gold standard test called the GH stimulation test is non-physiologic and has many shortcomings (1). They describe how an accurate diagnosis of GHD can be made using gene expression signatures in peripheral blood in a transcriptomic modality that combines gene expression data and random forest analysis. This new technique promises to increase the accuracy of diagnosing GHD and could potentially replace pharmacologic stimulation tests. Frontino et al. seek to expand the diagnostic criteria for Wolfram syndrome (WS) to include hypergonadotropic hypogonadism. WS is a rare autosomal recessive disease that presents classically with non-autoimmune diabetes mellitus, optic atrophy, diabetes insipidus, and deafness (2). These investigators report a higher prevalence of primary hypogonadism in children with WS and suggest that the increased occurrence of hypogonadism in WS warrants its inclusion in the diagnostic criteria to enable earlier detection and diagnosis. Wan et al. propose that an accurate diagnosis of disorders in sex differentiation (DSD) requires the detection of candidate genes as the genetic etiology of most individuals with DSD is unclear (3). These investigators report patients with DSDs whose whole-exome sequences identified a novel mutation that might signal via the upregulation of the β-catenin protein. These new diagnostic techniques will strengthen and possibly replace existing modalities.

The next set of articles focused on expanding the understanding of alterations in classic disease phenotypes through avenues such as the impact of COVID-19 on sexual maturation, emerging phenotypes in the diagnosis of insulinoma, reassuring findings on palpable breast tissue in infants, and the exciting impact of continuous glucose monitors (CGM) in non-diabetic conditions. Chioma et al. build on the hypothesis that the COVID-19 pandemic triggers pathological processes in humans (4, 5) to report a higher prevalence of rapidly progressive central precocious puberty (CPP) in girls during the COVID-19 pandemic from 2019 to 2022. Their finding that the prevalence declined after the peak of the pandemic suggests that the SARS-CoV-2 virus, or pandemic-associated environmental and psychosomatic changes could play a role in triggering CPP in girls. Melikyan et al., in another disease clarifying article, report an increased occurrence of multiple endocrine neoplasia type 1 (MEN 1) syndrome and Grade 2 tumors in children with insulinomas. The authors found that children with MEN 1 had a significantly higher number of pancreatic tumors when compared to those with sporadic insulinoma. Furthermore, family members of patients with MEN 1 had increased MEN 1 manifestations such as neoplasia of the parathyroid glands. They conclude that all children diagnosed with insulinoma should receive genetic testing, along with their family members, to exclude malignancies. They further recommend long-term follow-up of these patients. Another disease clarifying article from the Copenhagen Minipuberty Study explains that palpable breast tissue in infancy may be a benign process that does not require endocrinological investigation. This reassuring finding should prevent unnecessary evaluation in these infants and could lead to significant health care savings as the differential diagnosis of palpable breast tissue in infants ranges from benign physiological processes to severe pathologies such as hormone-secreting tumors (6). A close longitudinal follow-up will distinguish benign processes from other more serious conditions such as central precocious puberty and neoplasms. Buchanan et al. describe the first patient with an advanced bone age likely caused by elevated 11-ketotestosterone levels in Wiedemann-Steiner syndrome (WSS). This is important as the mechanism of advanced skeletal maturation is unclear in WSS (7). Sivasubramanian et al. examine the use of CGM in hyperinsulinemic hypoglycemia given that the use of CGM has revolutionized diabetes care and has enabled patients to monitor their glycemia in real time (8). They reported the feasibility and accuracy of CGM in children with hyperinsulinemic hypoglycemia. The safety and efficacy of this intervention in a non-diabetes-related field opens the field for the use of CGM for expanded indications such as in cystic fibrosis, glycogen storage diseases, and neonatal diabetes mellitus.

Nwosu’s new theory of hyperlipidemic memory of type 1 diabetes (T1D) is based on 4 clinical studies conducted in children, adolescents, and adults during their partial clinical remission (PR) phase of T1D. This theory explains the dichotomy in atherosclerotic cardiovascular disease risk between subjects with T1D who experienced PR, (remitters), and those who did not, (non-remitters) (9). This theory, which explains the lipid-based *macrovascular* complications of diabetes, complements the earlier theory of hyperglycemic memory which explains the glucose-based *microvascular* complications of T1D between the remitters and non-remitters. Nwosu’s hyperlipidemic memory theory and his concept of PR imprimatur have opened the field to inquiry into dyslipidemia in T1D based on PR history. It is conceivable that these inquiries could lead to changes in the guidelines for the prevention and management of dyslipidemia in T1D based on the observed dichotomy in lipid profile between the remitters and non-remitters.

In conclusion, the articles in this Research Topic will have lasting impact on the future of endocrinology. We thank all the Stars and Rising Stars in Endocrinology for their contributions.

## Author contributions

BN: Writing – review & editing, Writing – original draft, Visualization, Project administration, Methodology, Conceptualization.
